# Cisplatin-induced Pyroptosis Enhances the Efficacy of PD-L1 Inhibitor in Small-Cell Lung Cancer via GSDME/IL12/CD4Tem Axis

**DOI:** 10.7150/ijbs.89080

**Published:** 2024-01-01

**Authors:** Wendi Xuzhang, Tingting Lu, Weiqiu Jin, Yongfeng Yu, Ziming Li, Lan Shen, Xiaomin Niu, Xinghao Ai, Liliang Xia, Shun Lu

**Affiliations:** Shanghai Lung Cancer Center, Shanghai Chest Hospital, Shanghai Jiao Tong University School of Medicine, 241 Huaihai West Road, Shanghai 200030, People's Republic of China

**Keywords:** SCLC, Tumor immune microenvironment, Pyroptosis, GSDME, IL-12

## Abstract

The combination therapy of platinum-based chemotherapy and PD-L1 inhibitors but not the single anti-PD-L1 therapy has significantly improved the prognosis of patients with small-cell lung cancer (SCLC). However, the synergistic mechanism of combination therapy has not been fully elucidated. In this work, we identified a positive correlation between the expression of pyroptosis-related proteins Gasdermin E (GSDME) and the survival rates of patients with SCLC. Importantly, it was shown that human SCLC cell lines with high expression of GSDME showed more sensitivity to cisplatin, as well as cisplatin plus anti-PD-L1 treatment both *in vitro* and *in vivo*. Mechanically, cisplatin induced the activation of GSDME and the release of cytokines including IL-12, which enhance the expression of IFN-γ in T cells in the tumor immune microenvironment (TME) and subsequently improve anti-PD-L1 response. Altogether, our work demonstrates that cisplatin could induce GSDME-dependent cell pyroptosis to improve the response of anti-PD-L1 therapy though switching the TME from “cold” to “hot” in SCLC, indicating GSDME as a response biomarker for combination therapy of anti-PD-L1 and chemotherapy, as well as a potential target to sensitize the response to PD-L1 inhibitor therapy in future.

## Introduction

Small-cell lung cancer (SCLC) is an aggressive neuroendocrine malignancy known for its short doubling time, rapid growth rate, and early metastasis to distant sites 1. Around two-thirds of patients originally diagnosed with SCLC will develop extensive-stage SCLC (ES-SCLC). Platinum-based chemotherapy has been the primary treatment option for ES-SCLC over the past three decades. Nevertheless, despite its widespread use, the median overall survival (mOS) for ES-SCLC patients receiving platinum-based chemotherapy remains only 8-13 months 2-5. The prognosis of patients with ES-SCLC has been significantly improved by the use of immune checkpoint inhibitors (ICIs). The Phase III trial IMpower133 demonstrated that combining atezolizumab with carboplatin and etoposide in the first-line treatment regimen improved both overall survival (OS) and progression-free survival (PFS), representing a paradigm shift in the approach to ES-SCLC therapy 6. The Phase III study CASPIAN demonstrated that, in previously untreated patients with ES-SCLC, the combination of durvalumab and etoposide with either cisplatin or carboplatin significantly improved OS (13.0 months for ICIs combination therapy *vs*. 10.3 months for chemotherapy) 7. However, when used alone, nivolumab and pembrolizumab failed to show satisfactory results in subsequent studies 8-13.

Combination therapy, including immunotherapy in conjunction with first-line platinum-containing chemotherapy, has demonstrated value in the treatment of SCLC. Studies have shown that this approach is limited to improving overall response rate (ORR), PFS, and OS in patients with SCLC 9, 14, 15. Therefore, a comprehensive investigation into the mechanism by which chemotherapy enhances the effectiveness of ICIs is vital to predict and improve the efficacy of immunotherapy in SCLC. In non-small cell lung cancer (NSCLC), driver mutations, the expression levels of programmed cell death ligand 1 (PD-L1), and tumor mutational burden (TMB) have been identified as practical biomarkers for predicting the effectiveness of immunotherapy 16-22, however, widely acknowledged biomarkers in SCLC immunotherapy are still absent. Studies show that the SCLC-I subtype demonstrates a better response to ICIs compared to other subtypes (SCLC-A, SCLC-N, and SCLC-P). This subtype displays higher levels of CD8^+^ T cells, NK cells, macrophages, and B lymphocytes. Moreover, it highly expresses immune checkpoints such as PD-L1, programmed cell death protein 1 (PD-1), cytotoxic T-lymphocyte-associated antigen 4 (CTLA-4), cluster of differentiation 38 (CD38), indoleamine 2,3-dioxygenase 1 (IDO1), lymphocyte activation gene 3 (LAG3), and T-cell immunoglobulin and ITIM domain (TIGIT). Additionally, this subtype expresses human leukocyte antigen (HLA) at high levels. The SCLC-I subtype was validated in tumor samples from the IMpower-133 study and showed significantly greater survival benefits compared to the other three subtypes in patients who received immunotherapy combined with chemotherapy. Despite its ability to predict immune efficacy, the SCLC-I subtype's clinical application is challenging due to a lack of validation using real-world data and high cost. Since no confirmed predictive biomarkers exist, further research into new combination therapy options is necessary.

Pyroptosis refers to a form of programmed cell death that occurs through pro-inflammatory mechanisms 23. It is characterized by the formation of membrane pores, cellular swelling, rupture of the plasma membrane, and release of inflammatory substances. These characteristics distinguish pyroptosis from other forms of cell death, such as apoptosis 24, 25. In 2015, GSDMD was identified as a critical pyroptosis substrate. When cleaved by inflammatory caspases, GSDMD releases its N-terminal fragment, which can bind to lipids in the cell membrane, resulting in membrane perforation and an increase in osmotic pressure within cells. Gasdermin E (GSDME) is another member of the gasdermin family, located on human chromosome 7p15, that encodes 496 amino acids over ten exons 26. GSDME functions to regulate two forms of programmed cell death (PCD), apoptosis and pyroptosis 27-30. The GSDME protein is composed of two structural domains: the self-inhibitory C-terminal domain and the cytotoxic N-terminal domain 30. These two domains allow GSDME to maintain its conformational stability and regulate programmed cell death by either self-inhibition or induction of pyroptosis. Several studies have shown that chemotherapeutic drugs can activate GSDME, resulting in the stimulation of pyroptosis in cancer cells and the induction of antitumor immunity. Therefore, targeting GSDME is a promising strategy for the treatment of cancer.

GSDME-mediated pyroptosis can be triggered by both internal and external stimuli, including chemotherapy and some drugs associated with molecular-targeted therapy. These stimuli activate caspase-3, leading to cleavage of GSDME and resulting in inflammatory cell death 30. Studies have demonstrated that GSDME expression is also positively correlated with the phagocytosis of tumor-related macrophages, and with the production and function of NK cells and CD8^+^ T lymphocytes 31. We assumed that cisplatin-based chemotherapy might serve as an external stimulus that triggers GSDME-induced pyroptosis, which may play an essential role in enhancing the efficacy of anti-PD-L1 therapy in patients with SCLC.

## Materials and Methods

### Human specimens

Human SCLC specimens were obtained from the Shanghai Chest Hospital, Shanghai Jiao Tong University School of Medicine, in compliance with institutional guidelines for the use of human tissue in research. Only patients diagnosed with ES-SCLC were included. The patient information is summarized in Table 1.

### Cell culture and treatments

We acquired several cell lines from ATCC (USA), including SHP77, DMS114, GLC16, H69, and DMS53, all of which are human SCLC cells. The cells were cultured in RPMI or DMEM (Corning) supplemented with 10% fetal bovine serum (FBS, Gibco) and penicillin-streptomycin (Gibco). They were incubated at 37°C with 5% CO_2_ in a humidified incubator.

### Antibodies and reagents

To detect the target proteins, we used a set of antibodies, including anti-caspase-3 (CST), anti-GSDME (Abcam), anti-cleaved caspase-3 (CST), and anti-GAPDH (CST). Pyroptosis was induced by treating the cells with cisplatin (Selleck) in a serum-free medium overnight.

### cBioportal Dataset Acquisition

We obtained whole-genome sequences of 120 SCLC tumor samples and their paired normal tissues from the cBioportal public database (http://www.cbioportal.org/) based on specific screening criteria, such as a large sample size, authoritative research, and comprehensive and up-to-date data. The standard sequence number for the sequencing data was assigned as GSE69091, which was obtained from a study published in 2015^32^. For the secondary analysis of RNAseq data, we employed the GeneCards database to unify the matching and ID conversion processes.

### In vivo humanized NSG SCLC mouse model

Initially, on day 0, NSG (NOD.Cg-*Prkdc^scid^Il2rg^em1Sm^°^c^*, Shanghai Model Organisms) female mice (n = 5) were injected with 5 × 10^6^ PBMCs from human sources for 4-6 weeks through the tail vein to complete the humanized reconstruction. On the seventh day, human small cell lung cancer cells (5 × 10^6^ cells/mouse) were subcutaneously injected into the neck and back of humanized NSG mice. From around the 10^th^ day, cisplatin (8mg/kg) was administered every seven days; durvalumab (AstraZeneca, 10mg/kg), Recombinant Human IL-12 (huIL12, R&D, 100ng/kg), and human IL-12 affinity-purified polyclonal Ab (antiIL12, R&D, 500ng/kg) were administered every three days, respectively. Tumor volume was calculated using the following equation: tumor volume (mm^3^) = 0.5 × length (mm) × width mm^2^. Around the 40^th^ day, the mice were euthanized and the tumor and spleen were collected for further analysis. All mice were housed in a specific pathogen-free (SPF) Animal Center of Shanghai Chest Hospital. Animal experiments were approved by Shanghai Chest Hospital and performed according to the Laboratory regulations.

### Establishment of in vitro SCLC and PBMC co-culture model

For the Transwell assay, human small cell lung cancer cell lines were inoculated in the lower chamber, while human-derived PBMCs were inoculated in the upper chamber. The tumor cells and PBMCs were then co-cultured, and flow cytometry, RNA sequencing, RTCA, and other techniques were used to detect and analyze the PBMCs and tumor cells.

### Preparation of human peripheral mononuclear lymphocytes (PBMCs)

The collected anticoagulant blood was centrifuged to isolate peripheral blood cells. These cells were then resuspended in 6 mL of 1640 culture medium, repeatedly beaten and mixed, and subsequently added to 5 mL of Ficoll separation solution in a 1:1 volume ratio. The sample was centrifuged horizontally in slow lifting mode, and the resulting middle PBMC layer was gently aspirated and transferred to a centrifuge tube.

### Flow cytometry

The cells were collected and washed twice with PBS. They were then resuspended in FACS (PBS with 1% BSA) at a concentration of 1-5 x 10^6 cells/ml. The appropriate amount of fluorochrome-conjugated antibodies were added to the cells and incubated for 20-30 minutes at 4°C in the dark. The cells were washed twice with a staining buffer. Data was acquired using a flow cytometer (BD Biosciences). The experiment was conducted at least three times.

### Statistical analysis

All statistical analyses were carried out using GraphPad Prism8 (RRID:SCR_002798). Differences between groups were determined using the Student's t-test, and data are presented as mean ± SEM of at least three independent experiments. Statistically significant differences were defined as those with a P-value < 0.05.

## Results

### SCLC patients with high expression of GSDME in tumor cells show a more favorable prognosis

**Figure 1:**
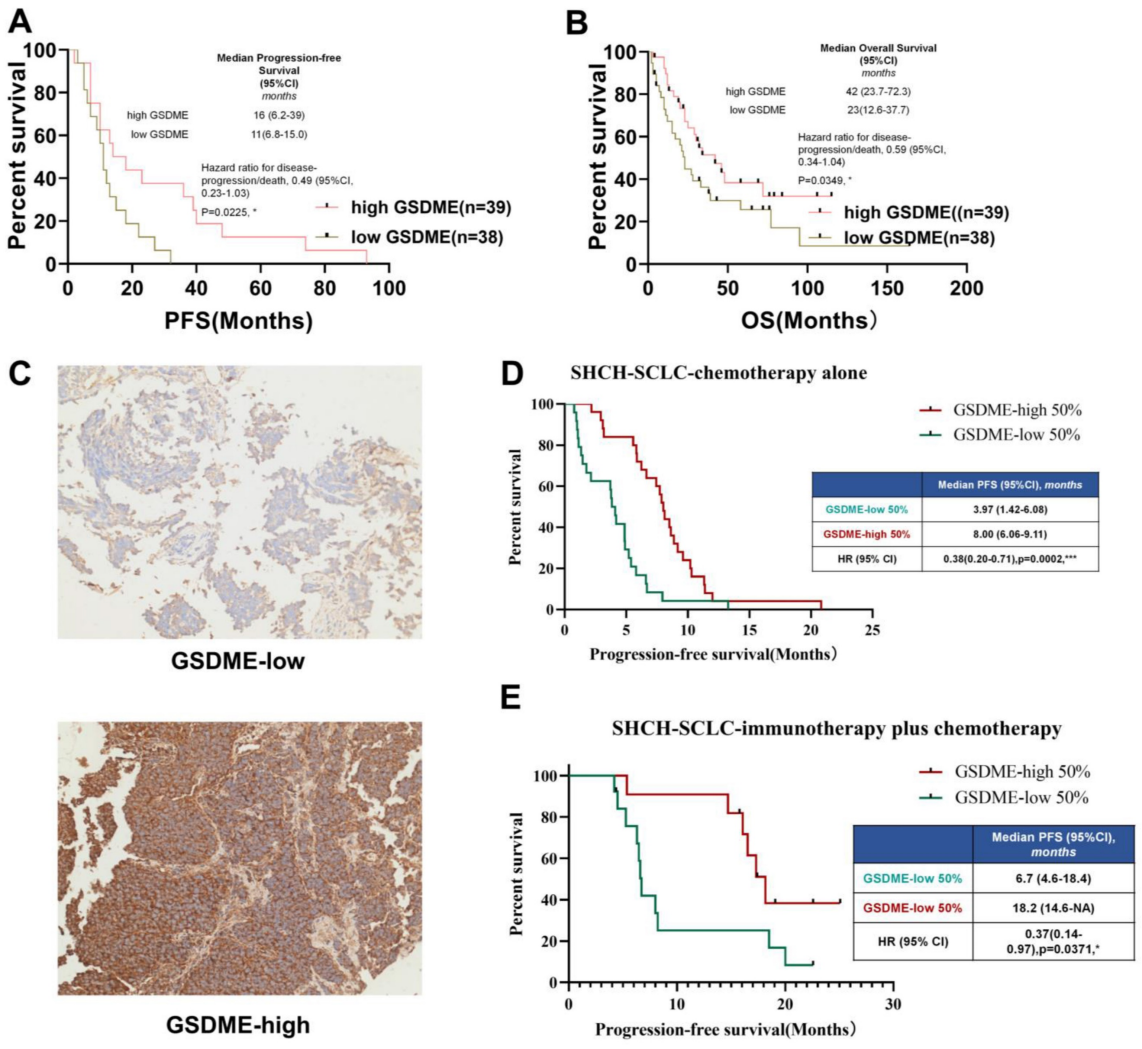
** SCLC patients with high expression of GSDME in tumor cells have better prognosis.** (A) PFS of GSDME^hi^ and GSDME^lo^ SCLC patients receiving chemotherapy (Data from cBioportal database). (B) OS of patients with GSDME^hi^ and GSDME^lo^ in SCLC patients receiving chemotherapy. (C) Representative images of immunohistochemical staining tumors from GSDME^hi^ and GSDME^lo^ SCLC patients. (D) PFS of GSDME^hi^ and GSDME^lo^ SCLC patients receiving chemotherapy (Data from Shanghai Chest Hospital). (E) PFS of patients with GSDME^hi^ and GSDME^lo^ SCLC patients receiving chemotherapy plus immunotherapy (Data from Shanghai Chest Hospital).

To determine the relationship between key molecules related to pyroptosis and the prognosis of chemotherapy in patients with SCLC, the cBioportal public database was first used to download RNA expression data of 120 patients with SCLC. We investigated the relationship between the mRNA expression levels of GSDMB, GSDMD, GSDME, and PFS of patients with SCLC who underwent chemotherapy. The results showed that the level of expression of GSDMB and GSDMD mRNA was not significantly related to the chemotherapeutic PFS of SCLC patients (Supplementary Figures 1A-B), but SCLC patients with high expression of GSDME had higher PFS (P = 0.0225, Figure 1A) and OS (P = 0.0249, Figure 1B).

To further verify whether the protein level of GSDME matched its mRNA level, paraffin tissues obtained from SCLC patients in our hospital stored since 2015 (n = 73, Table 1) were collected and immunohistochemical staining of GSDME was performed. The patients were divided into GSDME-low 50% and GSDME-high 50% groups based on the median H-score (Figure 1C). The Kaplan-Meier curve (Figure 1D) showed that the PFS of patients with SCLC in the GSDME-high 50% group receiving chemotherapy was significantly higher than the PFS of the GSDME-low 50% group (8.00 *vs*. 3.97 months; HR:0.38; 95% CI:0.20 to 0.71; P = 0.0002). The PFS of the GSDME-high 50% group of patients with SCLC who were administered immunotherapy combined with chemotherapy was significantly better than those of the patients in the GSDME-low 50% group (18.20 *vs*. 6.70 months; HR:0.37; 95% CI:0.14 to 0.97; P = 0.0371) (Figure 1E).

In SCLC patients receiving chemotherapy, GSDME expression was positively correlated with longer PFS (y = 2.0311x+94.947, R ² = 0.3906, two-tailed P < 0.0001) (Supplementary Figure 1C). For SCLC patients receiving chemo-immunotherapy, the expression of GSDME protein was positively correlated with longer chemotherapy PFS (y = 3.2745x+188.41, R ² = 0.3071, two-tailed P < 0.01) (Supplementary Figure 1D). These results suggest that GSDME may be a reliable predictive biomarker for SCLC chemotherapy alone and immunotherapy.

### SCLC patients with high expression of GSDME exhibited an immune-hot tumor microenvironment

**Figure 2:**
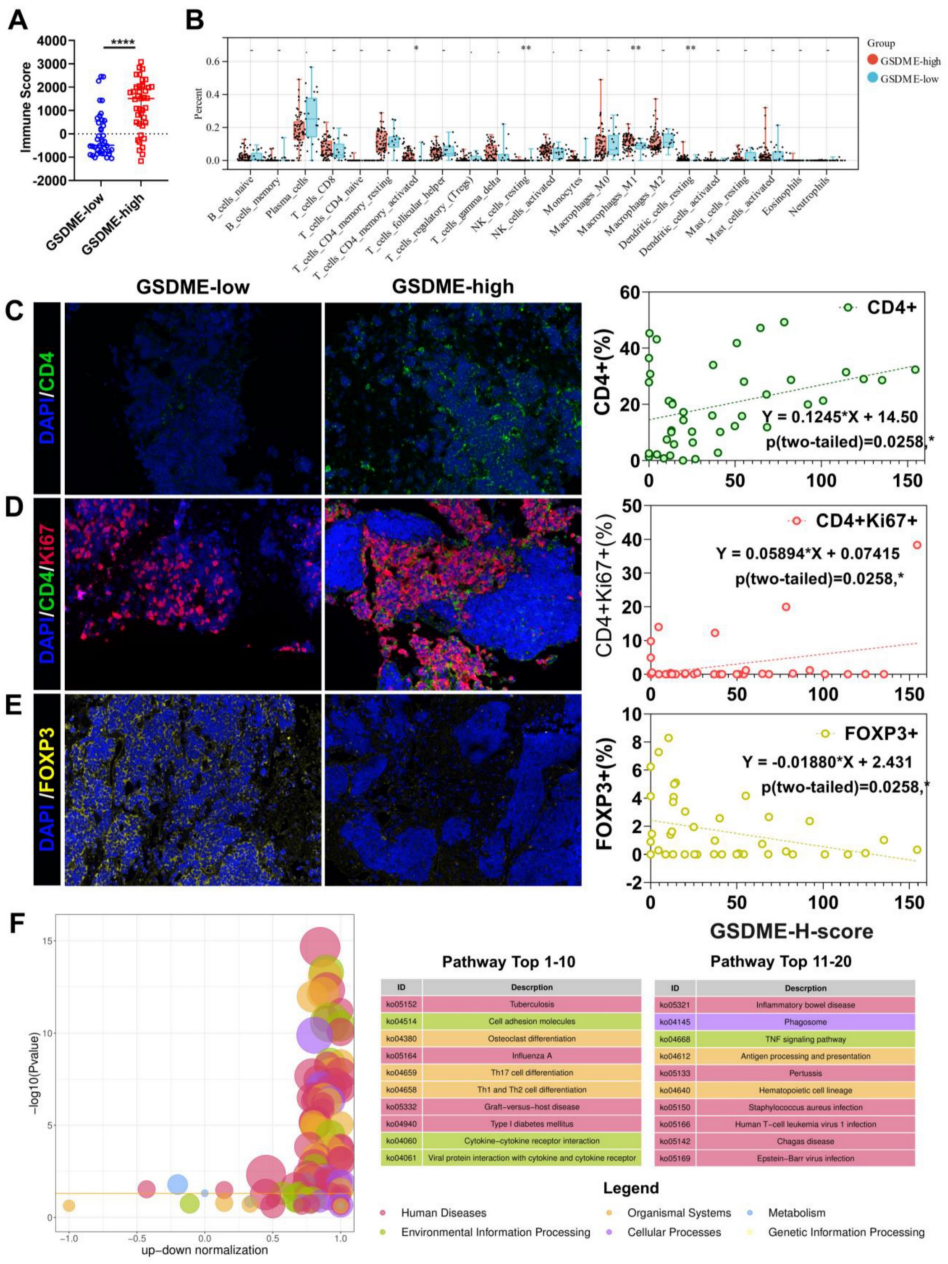
** The immune microenvironment of SCLC patients with high expression of GSDME is more similar to "hot tumor".** (A) ImmuneScore of patients with GSDME^hi^ and GSDME^lo^ SCLC patients (Data from cBioportal database). (B) CIBERSORT analysis compared 22 immune cell classifications between the GSDME^hi^ and GSDME^lo^ SCLC patients (Data from cBioportal database). (C) Immunofluorescence, representative staining images of CD4^+^ cells in the GSDME^hi^ and GSDME^lo^ groups, and pearson correlation was performed (Data from Shanghai Chest Hospital). (D) Immunofluorescence, representative staining images of CD4^+^Ki67^+^ cells in the GSDME^hi^ and GSDME^lo^ groups, and pearson correlation was performed (Data from Shanghai Chest Hospital). (E) Immunofluorescence, representative staining images of FOXP3^+^ cells in the GSDME^hi^ and GSDME^lo^ groups, and pearson correlation was performed (Data from Shanghai Chest Hospital). (F) Enrichment differential bubble plot, significantly up-regulated top 20 KEGG pathways in the GSDME^hi^ group than GSDME^lo^.

The response rate to cancer treatment is often closely linked to the tumor immune microenvironment (TME). To determine whether GSDME expression influences the TME, we divided patients into GSDME-high and GSDME-low groups based on their mRNA levels. ESTIMATE analysis showed that the immune score was significantly higher in the GSDME-high group (Figure 2A, Supplementary Figure 1E). Additionally, CIBERSORTx deconvolution analysis using R software revealed that CD4^+^ effector memory T cells, M1 macrophages, and resting DCs were substantially increased in the GSDME-high group compared to the GSDME-low group (Figure 2B). These findings were subsequently validated in our cohort, where we observed that the GSDME H score was positively correlated with the proportion of CD4^+^ T cells in SCLC (Y = 0.1245 * X+14.50, P = 0.0208) (Figure 2C) and the proportion of CD4^+^Ki67^+^ T cells (Y = 0.05894 * X+0.07415, P = 0.0290) (Figure 2D). Furthermore, the GSDME H score was negatively correlated with the proportion of regulatory T cells (Tregs) in SCLC (Y = -0.01880 * X+2.431, P = 0.0258) (Figure 2E). After performing differential gene analysis using the Limma method, we identified a significantly enriched cytokine-cytokine receptor interaction pathway (ko04060) in the GSDME-high group through KEGG enrichment analysis (Figure 2F).

HLA-related genes and interferon-γ-related T cell genes indicated that a better response to ICIs was significantly upregulated in the SCLC-I type 40, indicating the greater efficacy of ICIs. Similarly, in the GSDME-high group, we observed significant upregulation of HLA-related genes and 18 interferon-γ-related T cell genes (Supplementary Figure 1F). Further analysis of gene expression data in the database revealed significantly higher expression of immune checkpoint molecules (PDCD1LG2, LAG-3, and TIGIT), cell chemokines (CXCL9, CCL5, and CXCR6), and antigen presentation-related molecules (HLA) in the GSDME-high group (Supplementary Figure 1G). These findings suggest a strong correlation between pyroptosis and antitumor immunity. Consequently, SCLC patients with high GSDME expression possess greater immune infiltration and resemble the SCLC-I type, traits that may predict the efficacy of chemotherapy and immunotherapy.

Our analysis of public databases and specimens from our hospital revealed that patients with high GSDME expression had significantly improved PFS and OS compared to those with low GSDME expression. Furthermore, we found that the TME of GSDME-high patients was more similar to that of “hot tumor” compared to GSDME-low patients.

### The antitumor effects of GSDME depended on the immunocompetent system

**Figure 3:**
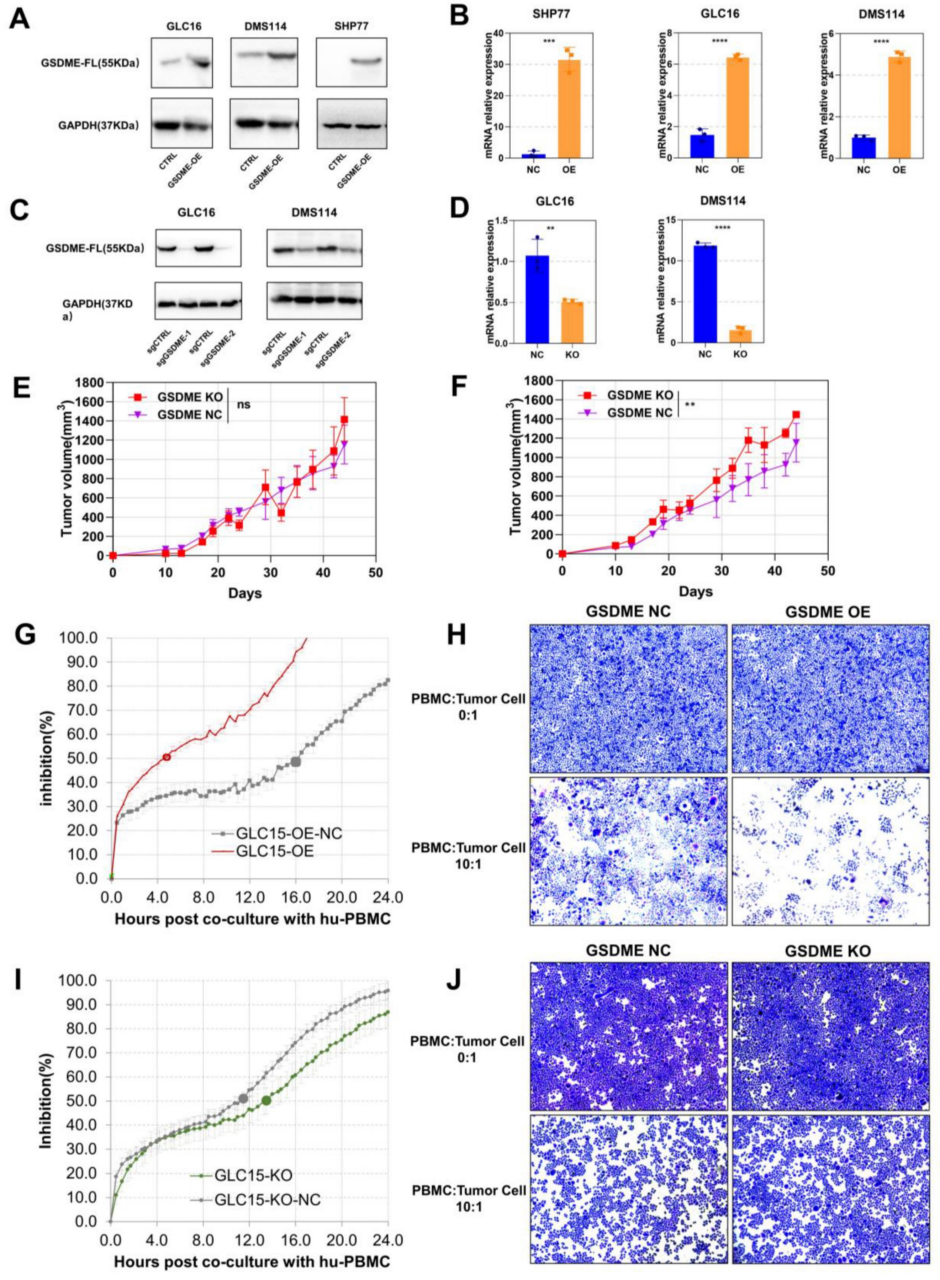
** The antitumor effects of GSDME depends on immunocompetent system.** (A) Overexpression of GSDME in small cell lung cancer cell lines were measured via western blot. (B) Overexpression of GSDME in small cell lung cancer cell lines were measured via RT-qPCR. (C) Knockout of GSDME in small cell lung cancer cell lines were measured via western blot. (D) Knockout of GSDME in small cell lung cancer cell lines were measured via RT-qPCR. (E) The growth curve of GSDME-KO and GSDME-NC NSG mice bearing tumors (DMS114). (F) The growth curve of GSDME-KO and GSDME-NC humanized NSG mice bearing tumors (DMS114). (G) Inhibition rate of GSDME-OE human SCLC cell lines and their GSDME-NC group when co-cultured with human PBMC via RTCA. (H) Crystal violet staining image of live GSDME-OE human SCLC cell lines and their GSDME-NC group co-cultured with human PBMC. (I) Inhibition rate of GSDME-KO human SCLC cell lines and their GSDME-NC group when co-cultured with human PBMC via RTCA. (J) Crystal violet staining image of live GSDME-KO human SCLC cell lines and their GSDME-NC group co-cultured with human PBMC.

To investigate the function of GSDME in SCLC, we conducted western blotting assays to determine the baseline expression of GSDME in seven human SCLC cell lines (Supplementary Figure 2A). We selected cell lines with low GSDME expression to construct overexpressed cell lines (GSDME-OE), while cell lines with high GSDME expression to create knockout cell lines (GSDME-KO). Overexpression and knockout of GSDME were validated at both transcription level and expression level through real-time fluorescent quantitative PCR (qRT-PCR) and western blotting, respectively (Figure 3A-D).

We implanted GSDME-NC and GSDME-KO cells into the neck and back of NSG mice at a concentration of 5 × 10^6^ cells per mouse. Interestingly, there was no significant difference in the size of subcutaneous tumors formed by GSDME-NC and GSDME-KO cells in NSG mice (Figure 3E), which lack T, B, and NK cells. However, in humanized-NSG mice (Supplementary Figure 2C), GSDME-KO tumors were significantly larger than GSDME-NC tumors (Figure 3F). Thus, our findings suggest that GSDME expression does not directly affect the proliferation of SCLC cells. Instead, the comparison between humanized-NSG mice and NSG mice indicates that the antitumor effect of GSDME is heavily influenced by the immunocompetent system.

We co-cultured human SCLC cells with human PBMC *in vitro* and monitored the process in real-time using the real-time cell analysis (RTCA) technology (Supplementary Figure 2B). Crystal violet was used to stain live tumor cells (PBMC: tumor cell ratio = 10:1) after 48 hours of co-culture. RTCA demonstrated that GSDME-OE cells showed half inhibition at 4.7 hours, while the control group reached half inhibition at 16.0 hours (Figure 3G). Similarly, GSDME-KO cell co-cultured with human PBMC showed half inhibition at 13.5 hours, whereas the NC group achieved this at 11.5 hours (Figure 3I). The crystal violet staining results aligned with those observed for RTCA (Figures 3H and 3J). Our findings indicate that the overexpression or knockout of GSDME does not affect the proliferation of SCLC cell lines in the absence of immune cells, which is consistent with our *in vivo* results. However, the overexpression of GSDME enhanced the ability of PBMCs to kill SCLC cells, while knocking out GSDME impaired it. Hence, GSDME exerts its antitumor effect in SCLC through its interaction with the immune system, rather than by affecting tumor proliferation.

### Chemotherapy induced GSDME-mediated pyroptosis and the overexpression of GSDME significantly increased cisplatin sensitivity

**Figure 4:**
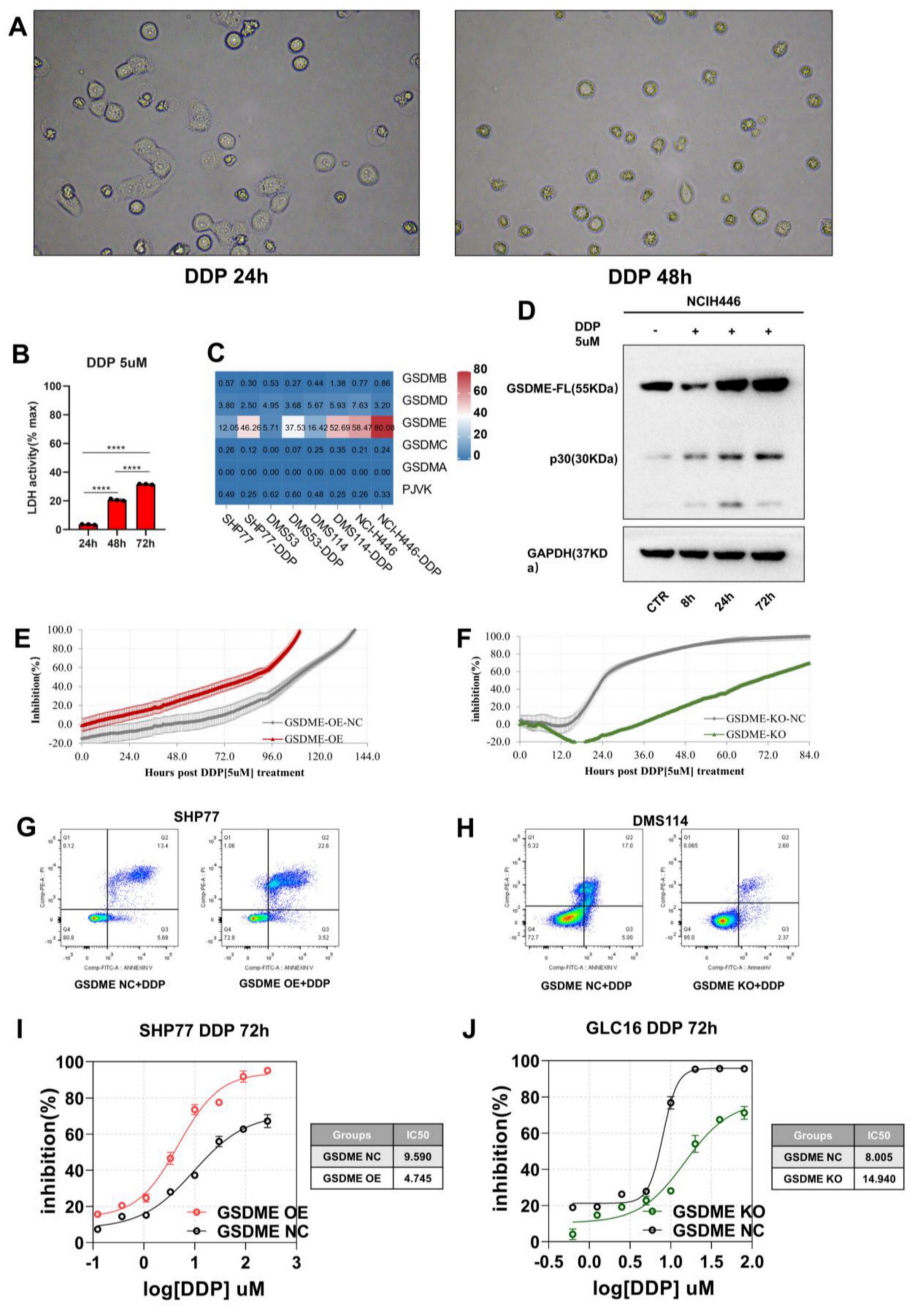
** Chemotherapy induces GSDME mediated pyroptosis, and overexpression of GSDME significantly enhances cisplatin sensitivity.** (A) Bright-field images of SCLC cell lines treated with 5 μM cisplatin for 24 and 48 hours via phase-contrast microscope. (B) LDH release activity was detected after 5 μM cisplatin after 24, 48 and 72 hours, respectively. (C) RT qPCR was used to detect the mRNA expression level of Gasdermin family proteins in SCLC cell lines treated with 5 μM cisplatin. (D) Western blot showed that 5 μM cisplatin was applied to small cell lung cancer cell lines, and the expression levels of GSDME full-length protein and GSDME-N-terminal protein were detected at 0, 8, 24 and 72 hours, respectively. (E) RTCA displays an inhibition rate of 5 μM cisplatin in GSDME-OE and GSDME-NC GLC16 cell lines. (F) RTCA displays an inhibition rate of 5 μM cisplatin in GSDME-KO and GSDME-NC GLC16 cell lines. (G) The degree of cell death in SHP77 GSDME-OE cell and GSDME-NC group after 5 μM cisplatin for 24 hours via Annexin V/PI. (H) The degree of cell death in DMS114 GSDME-KO cell and GSDME-NC group after 5 μM cisplatin for 24 hours via Annexin V/PI. (I) CCK8 showed a curve fitting the inhibition rate 72 hours after the gradient concentration of cisplatin acted on SHP77 GSDME -OE and GSDME-NC group. (J) CCK8 showed a curve fitting the inhibition rate 72 hours after the gradient concentration of cisplatin acted on GLC16 GSDME -KO and GSDME-NC group.

Given that cisplatin is the standard chemotherapy for SCLC patients, we first treated the SCLC cell line with cisplatin (DDP). Using a phase-contrast microscope, we observed pyroptosis in the SCLC cell line and noted an increasing number of cells with balloon-like characteristics over time (Figure 4A). Our qPCR analysis revealed a significant increase in the expression of GSDME mRNA without the activation of other gasdermin family molecules (Figure 4C). Additionally, we observed a time-dependent increase in GSDME-N terminal expression (cleaved fragment) (Figure 4D) and LDH release (Figure 4B).

GSDME expression is closely related to cisplatin sensitivity in non-small cell lung cancer. We performed RTCA and Cell Counting Kit 8 (CCK8) assays to monitor the inhibitory effect of cisplatin on SCLC cell growth. To evaluate the degree of cisplatin-induced cell death, we used flow cytometry with Annexin V/PI staining. Under cisplatin induction (5 µM), GSDME-OE cells reached a half inhibition rate at 85 hours, while GSDME-OE-NC cells required 108 hours to achieve it (Figure 4E). Furthermore, the median inhibitory concentration (IC_50_) of cisplatin for inhibiting GSDME-OE SHP77 cells was 4.745 µM, while the IC_50_ of the NC group was 9.590 µM, according to CCK8 results (Figure 4I). Annexin V/PI staining revealed a higher proportion of advanced apoptosis in the SHP77-OE group than in the control group (Figure 4G). Our results suggest that GSDME overexpression can significantly enhance the sensitivity of SCLC cells to cisplatin *in vitro*, ultimately promoting cell death.

In contrast to the GSDME-OE cells, GSDME-KO cells required 68 hours to reach half inhibition, while GSDME-KO-NC cells needed only 24 hours to achieve half inhibition (Figure 4F). According to CCK8 results, the IC_50_ of cisplatin for GSDME-KO cells was 14.940 µM, while that of the control group was 8.005 µM (Figure 4J). Annexin V/PI staining revealed a lower proportion of apoptotic cells in the GSDME-KO group than in the control group (Figure 4H). Thus, our results suggest that knocking out GSDME significantly reduces the sensitivity of SCLC cells to cisplatin *in vitro*, which significantly decreases cell death. Overall, GSDME overexpression significantly enhances the cisplatin sensitivity of SCLC, while knocking out GSDME significantly inhibits it. In summary, the antitumor effects of GSDME in SCLC require induceable stimuli, whether exogenous or endogenous.

### The expression of GSDME regulated the cisplatin-activated IL12RB1-IL12 pathway

To further investigate how GSDME affects the efficacy of cisplatin, we treated GSDME-OE, GSDME-KO, and their respective NC groups with cisplatin. We then conducted RNA-seq analysis to explore the underlying mechanisms.

**Figure 5:**
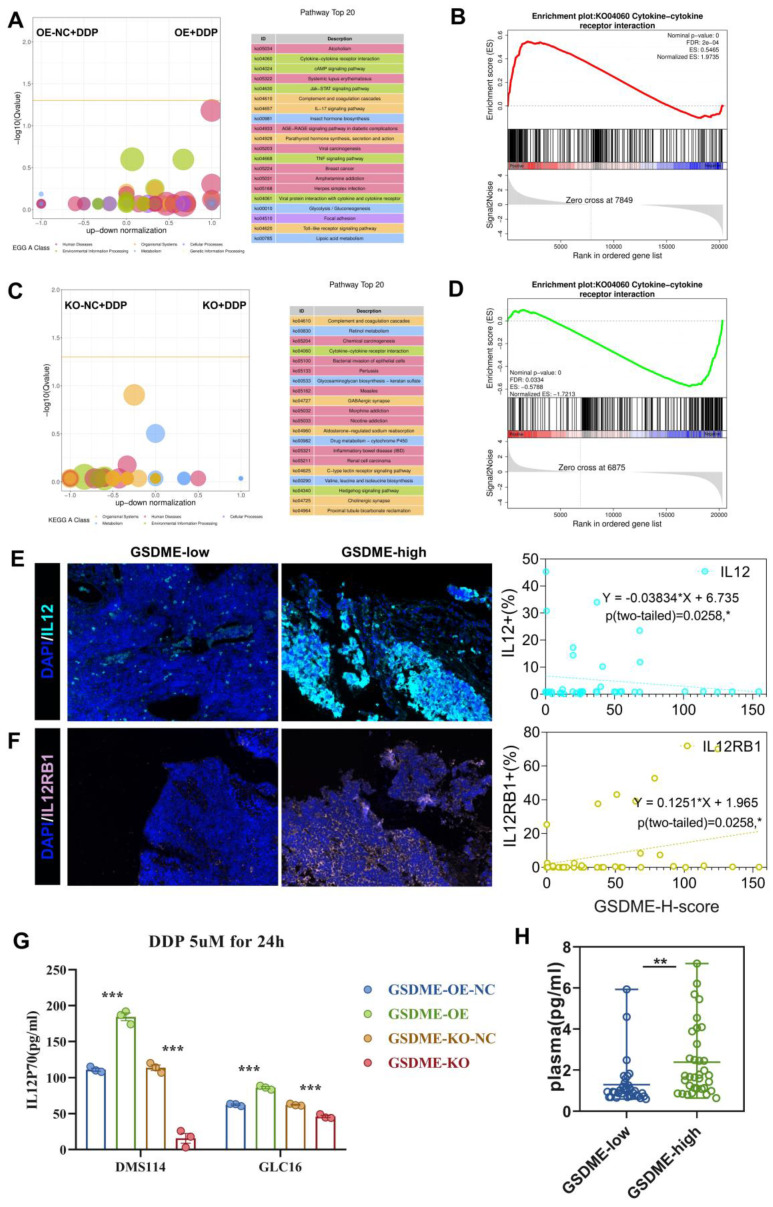
** The expression of GSDME regulates the cisplatin activated IL12RB1-IL12 pathway.** (A) Enriched differential bubble plot, showing the top 20 KEGG pathways significantly upregulated in the GSDME-OE group compared to GSDME-NC group after cisplatin induction. (B) GSEA plot reveals the regulatory effect of Cytokine-Cytokine receptor interaction (ko04060) in the GSDME-OE group and GSDME-NC after cisplatin induction. (C) Enriched differential bubble plot, showing the top 20 KEGG pathways significantly upregulated in the GSDME-KO group compared to GSDME-NC group after cisplatin induction. (D) GSEA plot reveals the regulatory effect of Cytokine-Cytokine receptor interaction (ko04060) in the GSDME-KO group and GSDME-NC after cisplatin induction. (E) Immunofluorescence, representative staining images of IL-12^+^ cells in the GSDME^hi^ and GSDME^lo^ groups, and pearson correlation was performed (Data from Shanghai Chest Hospital). (F) Immunofluorescence, representative staining images of IL12RB1^+^ cells in the GSDME^hi^ and GSDME^lo^ groups, and pearson correlation was performed (Data from Shanghai Chest Hospital). (G) The concentration of IL-12P70 in the cell culture supernatant of GSDME-OE/KO and GSDME-NC SCLC lines induced by cisplatin via ELISA. (H) Luminex detection of plasma IL-12P70 concentrations in patients with extensive stage small cell lung cancer in the GSDME high expression group and GSDME low expression group. (I) The concentration of plasma IL-12P70 in GSDME-high and GSDME-low patients with ES-SCLC via Luminex.

Upon treating cells with 5 µM cisplatin, the KEGG enrichment differential bubble plot revealed that the cytokine-cytokine receptor interaction (ko04060), JAK-STAT signaling pathway (ko04630), IL-17 signaling pathway (ko04657), and TNF signaling pathway (ko04668) were significantly upregulated in the GSDME-OE group (Figure 5A). Additionally, GSEA analysis showed that cytokine-cytokine receptor interaction (ko04060) was significantly upregulated in this group (nominal P-value <0.0001, Figure 5B). The corresponding volcano plot (Supplementary Figure 3A) highlighted the significant upregulation of specific genes, including IL12RB1, in the cytokine-cytokine receptor interaction (ko04060) pathway in the GSDME-OE group. In contrast, the KEGG enrichment differential bubble plot demonstrated that the complement and coagulation cascade (ko04610) and cytokine-cytokine receptor interaction (ko04060) pathways were significantly downregulated in the GSDME-KO group upon treatment with 5 µM cisplatin (nominal P-value <0.0001, Figure 5C-D). Moreover, the expression of IL12A and IL12RB1 was downregulated in the GSDME-KO group (Supplementary Figure 3B).

Our RNA-seq analysis revealed that GSDME increased cisplatin sensitivity and was closely associated with the cytokine-cytokine receptor interaction (ko04060) pathway, consistently indicating possible tumor immunity mediation by GSDME through the regulation of this pathway. This mechanism could lead to the inhibition of SCLC proliferation and promotion of pyroptosis. Quantitative immunofluorescence analysis further confirmed the positive correlation between the GSDME-H-score and the expression of IL-12RB1 and IL-12 (Figures 5E-F). *In vitro*, our findings reveal that the GSDME-OE group exhibited a noticeable increase in the active component of IL-12, IL-12P70, in the cell supernatant induced by cisplatin, whereas the GSDME-KO group showed a significant decrease (Figure 5G). Not only that, but we also conducted a thorough analysis of SCLC patients' plasma, and the results indicate that GSDME^hi^ SCLC patients had notably higher levels of IL12P70 (2.33 pg/mL *vs.* 1.30 pg/mL, P = 0.0031) (Figure 5H).

### Overexpression of GSDME significantly increased the efficacy of chemo-immunotherapy

To establish a humanized NSG mouse model of SCLC, we injected 5×10^6^ huPBMCs into the tail vein of female NSG mice (4-6 weeks old) on day 0. Subsequently, we subcutaneously inoculated human SCLC cell lines (5×10^6^ cells per mouse) into the neck and back of these humanized NSG mice on day 7. Beginning on day 10, we administered the drug as per the experimental requirements and monitored the size of the subcutaneous tumors every three days. Around day 40, we euthanized the mice and collected the tumor spleens for further analysis (Supplementary Figure 2C).

Our findings revealed that patients with GSDME^hi^ who underwent chemo-immunotherapy had significantly longer PFS compared to GSDME^lo^ patients with GSDME^lo^. As we discovered earlier, GSDME mediates antitumor immunity and releases IL-12 by regulating the cytokine-cytokine receptor interaction (ko04060) pathway. Some clinical trials have shown that IO monotherapy cannot improve the survival of SCLC patients. Based on this information, we hypothesized that chemotherapy activates GSDME, inducing SCLC pyroptosis and releasing IL-12, which in turn reshapes the TME, promoting the anti-PD-L1 effect.

Chemotherapy combined with anti-PD-L1 antibody therapy is the first-line treatment for SCLCs. Whether GSDME can serve as a key molecule in altering the efficacy of chemotherapy and PD-L1 inhibitors needs to be investigated.

**Figure 6:**
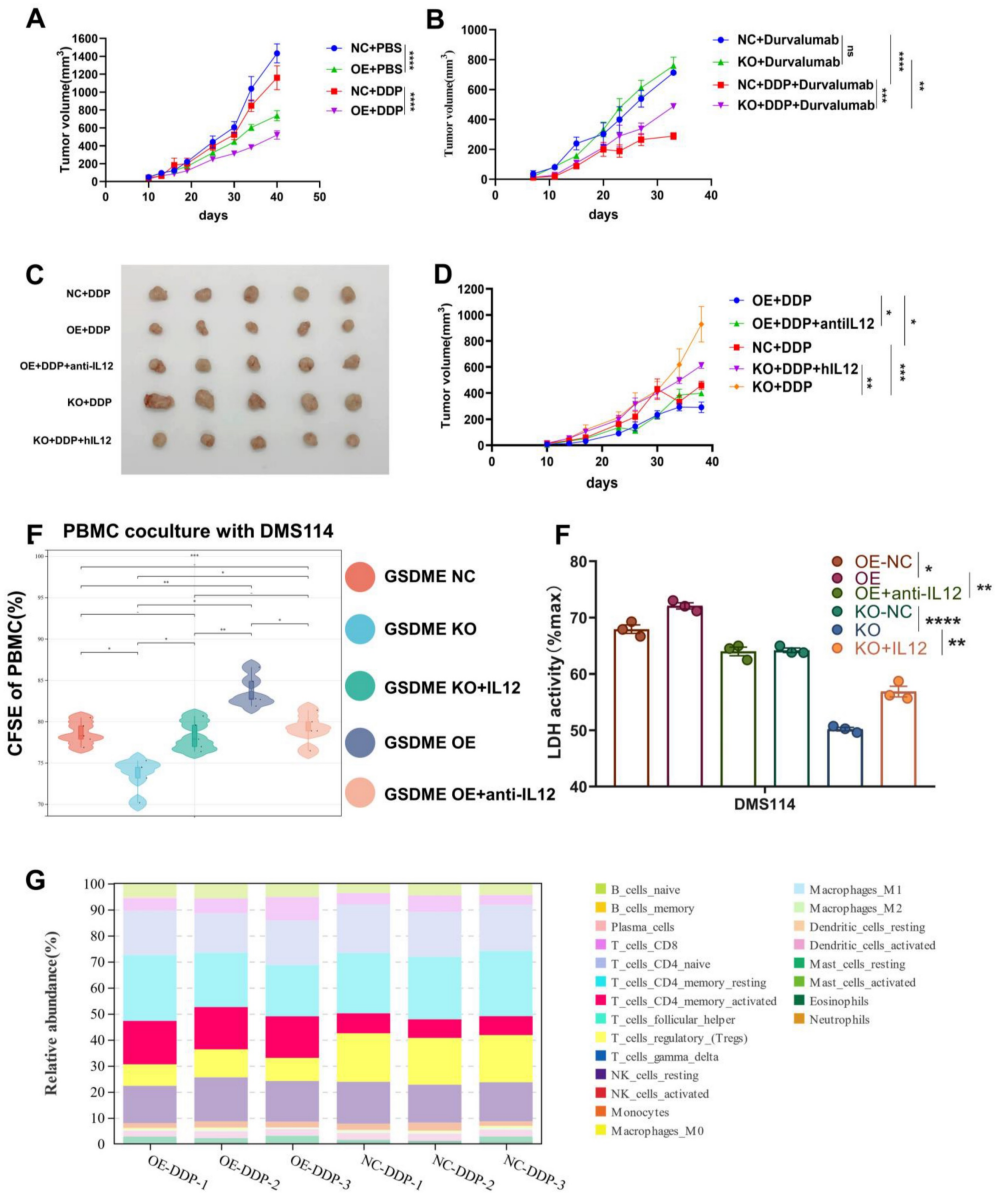
** Overexpression of GSDME significantly increases the efficacy of chemo-immunotherapy.** (A) The growth curve of GSDME-OE and GSDME-NC humanized NSG SCLC mice treated with cisplatin (GLC16). (B) The growth curve of GSDME-OE and GSDME-NC humanized NSG SCLC mice treated with cisplatin and/or Durvalumab (GLC16). (C) Tumor size of GSDME-OE/KO and GSDME-NC humanized SCLC NSG mice. The GSDME-KO group received human recombinant IL-12, while the GSDME-OE group received human recombinant neutralizing IL-12 protein intravenously. (D) The growth curve of GSDME-OE/KO and GSDME-NC humanized SCLC NSG mice. The GSDME-KO group received human recombinant IL-12, while the GSDME-OE group received human recombinant neutralizing IL-12 protein intravenously. (E) In vitro, PBMC was co-cultured with GSDME-OE/KO and GSDME-NC DMS114 cells, and treated with cisplatin. Recombinant IL-12 and neutralizing IL-12 protein was administered in GSDME-KO and GSDME-OE group respectively. PBMC was collected for proliferation test via CFSE. (F) In vitro, GSDME-OE/KO and GSDME-NC DMS114 cells were co-cultured with PBMC, and treated with cisplatin. Recombinant IL-12 and neutralizing IL-12 protein was added to GSDME-KO and GSDME-OE group respectively. Cell killing ability was quantified by LDH release from the co-culture supernatant. (G) CIBERSORT analyzed the 22 immune cell ratios of tumors from GSDME-OE and GSDME-NC humanized SCLC NSG mice ungergoing cisplatin induction.

In the humanized NSG mouse model, we observed a significant reduction in the size of SHP77 subcutaneous tumors in the GSDME-OE group compared to those in the GSDME-NC group following cisplatin treatment (Figure 6A). This finding suggests that GSDME overexpression can markedly enhance the inhibitory effect of cisplatin on tumors *in vivo*. As wild-type SHP77 does not express GSDME, our results strongly suggest that the antitumor effect of chemotherapy in SCLC partially relies on GSDME. In contrast, monotherapy with durvalumab (anti-PD-L1) did not produce a significant difference in tumor size between the GSDME-KO and GSDME-NC groups (Figure 6B), mirroring the repeated failure of PD-L1 inhibitor monotherapy in SCLC clinical settings. Similarly, overexpression of GSDME alone did not enhance the efficacy of PD-L1 immunotherapy (Supplementary Figure 5). However, when we combined chemotherapy with durvalumab treatment, subcutaneous tumors in the GSDME-KO group were noticeably larger than those in the GSDME-NC group (Figure 6B). These support the idea that GSDME needs to be induced by cisplatin to have a significant anti-tumor effect.

We collected humanized NSG mouse tumors and utilized bulk RNA-Seq and CIBERSORT deconvolution to analyze them. After cisplatin treatment, we noticed a significantly higher proportion of CD4 effector memory T cells in GSDME-OE tumors compared to that in the control group, while the proportion of regulatory T cells was markedly lower than the control group (Figure 6G). These results were consistent with human SCLC data (Figure 2B), indicating that GSDME could play an antitumor role after cisplatin induction by activating CD4 effector memory T cells and inhibiting regulatory T cells. Following cisplatin induction, the tumor immune microenvironment of SCLC overexpressing GSDME tended to become “hotter” than that in the NC group.

The *in vivo* phenotypic results strongly supported our hypothesis that chemotherapy induced GSDME-dependent cell death, which subsequently led to the reshaping of TME, resulting in improved efficacy of PD-L1 inhibitors.

### IL-12 rescue reduced efficacy of chemotherapy caused by the deficiency of GSDME

We observed that GSDME overexpression released higher levels of the active component IL-12 compared to the NC group after cisplatin induction, while GSDME knockout had the opposite effect. IL-12 plays a vital role in antitumor immunity. Thus, we carried out further investigations to determine whether exogenous recombinant human IL-12 restored antitumor activity in GSDME-deficient SCLC and whether neutralizing IL-12 antibodies could inhibit antitumor function in the GSDME-OE group.

*In vivo*, we observed that GSDME-OE SCLC subcutaneous tumors were noticeably smaller than those in the GSDME-NC group. However, the concurrent administration of neutralizing IL-12 antibodies reduced this antitumor effect. Conversely, GSDME-KO SCLC subcutaneous tumors were substantially larger than those in the NC group, and the simultaneous administration of IL-12 caused tumor shrinkage (Figures 6C and 6D).

We used CFSE (carboxyfluorescein succinimidyl amino ester) to evaluate the proliferation ability of PBMCs co-cultured with the GSDME-KO group after cisplatin induction and observed a significant decrease in this ability. However, this effect was restored upon the addition of exogenous recombinant IL-12. Conversely, the proliferative ability of PBMCs co-cultured with the GSDME-OE group was enhanced, which was reversed by the addition of recombinant neutralizing IL-12 antibodies (Figure 6E). After co-culture, we noticed a reduction in cell death in the GSDME-KO group compared to that in the GSDME-NC group, which was restored by the use of recombinant IL-12 as observed quantitatively by LDH release. In contrast, the GSDME-OE group exhibited increased cell death compared to the NC group, which was restored by the use of recombinant neutralizing IL-12 antibodies (Figure 6F). Baesed on the above results, we found that chemotherapy activates GSDME-dependent SCLC pyroptosis, releases IL-12, and reshapes the TME, thus promoting the efficacy of PD-L1 inhibitors.

### GSDME reshaped the cisplatin-induced SCLC TME through the IL12RB1-IL12-CD4 effector memory T cell pathway

To understand the mechanisms behind the enhanced therapeutic effect of PD-L1 after cisplatin induction due to GSDME overexpression, we carried out RNA-seq on PBMCs that had been co-cultured for over 48 hours. Our GO analysis revealed that the GSDME-OE group exhibited significant activation of cell killing, T cell migration, T cell chemotaxis, T cell proliferation, interleukin-12 production, the JAK-STAT cascade, CD4-positive alpha-beta T cell activation, interferon-gamma production, dendritic cell chemotaxis, and dendritic cell chemotaxis in PBMC (Supplementary Figure 4A), whereas an opposite trend was observed in the GSDME-KO group (Supplementary Figure 4B). These findings suggest that GSDME can reshape the TME by regulating the differentiation of T helper cells.

We conducted subsequent cluster analysis of PBMC sequencing data and observed that in the GSDME-OE-DDP group, molecules that promote Th1 differentiation such as IL-2, IFNG, IFNGR, IL12A, IL12R, and STAT4 were considerably upregulated, whereas molecules such as IL-17A, IL-17F, FOXP3, IL1B, TGFB, STAT3, and others that promote Th17 differentiation were notably downregulated (Supplementary Figure 4C). Furthermore, during co-culture with GSDME-OE cancer cells, we noticed that T cells tended to transform into CD4^+^ T effector memory cells.

To determine whether the effect is mediated by IL-12, we used flow cytometry and observed that the expression of GSDME in tumor cells did not affect the total number of CD4^+^ T cells *in vitro* (Supplementary Figure 4E-G). However, we noticed that neutralizing IL-12 antibodies had the ability to suppress Th1 cells, CD4^+^ effector memory T cells, and the increase in IFN-γ in the PBMCs of the GSDME-OE co-culture group (Supplementary Figure 4H-J). Furthermore, this reversed the decline in Tregs (Supplementary Figure 4K). Dendritic cells play a crucial role in antigen presentation, and we identified that activated DCs were substantially upregulated in the GSDME-OE group, in which IL-12P70 and IL-12RB1 were also upregulated. Nevertheless, these effects disappeared after the administration of exogenous neutralizing IL-12 antibodies (Supplementary Figure 4L-N).

**Figure 7:**
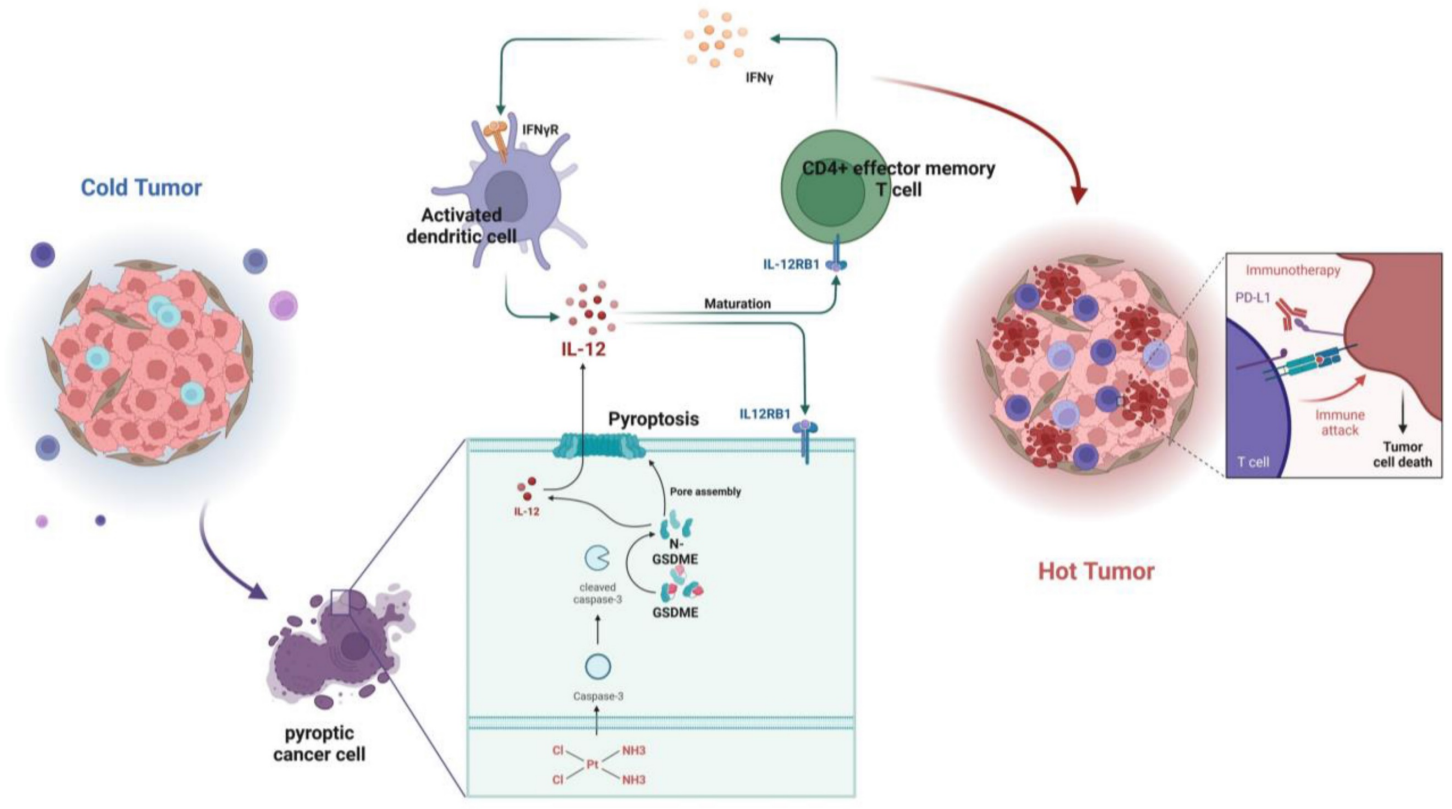
** Graphical Abstract.** In SCLC, GSDME improves the efficacy by reshaping the tumor microenvironment induced by cisplatin. GSDME-OE SCLC tumors activate the IL12RB1-IL12 pathway, release more active components of IL12, subsequently promote differentiation towards CD4 effector memory T cell. Thus, T cells were stimulated to release IFN-γ, so that dendritic cells were able to release more IL-12, transforming a vicious anti-tumor cycle. Exogenous IL-12 restores the weakened chemotherapy efficacy caused by knocking out GSDME, while neutralizing IL-12 antibodies restores the enhanced chemotherapy efficacy caused by overexpression of GSDME. In conclusion, GSDME reshapes the cisplatin-induced SCLC tumor microenvironment through the IL12RB1-IL12-CD4 effector memory T cell pathway, thereby improving the efficacy of chemo-immunotherapy.

In summary, GSDME plays a crucial role in sensitizing immunotherapy by reshaping the TME induced by cisplatin in SCLC. In the GSDME-OE SCLC tumors, the IL12RB1-IL12 pathway is activated, leading to the release of more active components of IL12, the activation of CD4 effector memory T cells, and stimulation of T cells to release IFN-γ. The activation of DCs by IFN-γ results in the release of more IL-12, forming a virtuous circle. Additionally, exogenous IL-12 restores the decrease in the efficacy of chemotherapy caused by GSDME knockout, while neutralizing IL-12 antibodies restore the enhanced chemotherapeutic efficacy caused by GSDME overexpression. Therefore, GSDME is responsible for reshaping the cisplatin-induced SCLC TME through the IL12RB1-IL12-CD4 effector memory T cell axis, thus improving the efficacy of chemo-immunotherapy (Figure 7).

## Discussion

This study presents a novel approach to creating a mouse model for small cell lung cancer (SCLC), which closely mimics the disease progression in humans. Unlike the traditional technique, which lacks TP53 and RB1 genes, this model has a humanized immune system and can be employed to investigate various subtypes of SCLC and even individual patients. This innovation is highly flexible and has the potential to replicate the clinical course of the disease with greater accuracy. For the first time, this study proposes that GSDME achieves its anti-tumor effect by regulating the interaction of IL-12RB1-IL-12, which has been demonstrated through recovery experiments. It was found that patients with high expression of GSDME have activated CD4^+^ effector memory T cells, inhibited regulatory T cells, and a relatively "hot" TME. This was confirmed *in vivo* experiments. Nonetheless, alternative pyroptotic pathways, such as the utilization of caspase1 to cleave GSDMD, have been found to facilitate IL1β maturation and release. Additionally, prior research has indicated that GSDME can induce cell pyroptosis independently of GSDMD. These findings imply that the dominant executing molecule of cell pyroptosis may vary across different tumors, underscoring the need for further exploration into their intricate interplay.

The relationship between GSDME and cancer was first discovered in 1998. Multiple studies have shown that in a wide range of estrogen receptor-positive (ER+) breast cancer cell lines, the level of GSDME expression is often low, indicating its possible role as a decisive factor in hormone-unresponsive breast cancer. Subsequently, more studies have revealed that epigenetic inactivation, caused by methylation, leads to lower expression of GSDME in most cancer cells as compared to normal cells 33-35. It is well known that DNA methylation is one of the characteristic manifestations of cancer, and the promoter of GSDME with dinucleotide-rich CpG island contributes to the methylation in cancer cells, where methylation silences the *gsdme* gene, providing fertile ground for the tumor growth 34, 36. Therefore, regulation of protein expression through methylation modification may be an important regulatory mechanism involved in tumor occurrence by GSDME.

Moreover, multiple *in vitro* and *in vivo* studies have confirmed the anti-tumor effects of GSDME. *In vitro* studies have demonstrated that overexpression of GSDME leads to a significant reduction in the proliferation and invasion abilities of cancer cells, whereas downregulation of GSDME enhances these abilities 33, 34, 37. In the colitis-associated colon cancer model, the number of tumors was observed to be significantly increased in *gsdme^-/-^* mice as compared to their wild-type littermates 38. In the melanoma mouse model, the results of tumor growth monitoring revealed that the tumor formation and growth rate of GSDME knockout tumors were significantly faster than those of tumors expressing GSDME 37. Loss of function mutations in GSDME, which are often associated with cancer, further support the notion that GSDME can function as a tumor inhibitor 31.

Previous studies have identified three key ways through which GSDME exerts its anticancer effects. First, GSDME-driven pyroptosis can be triggered by internal or external stimuli, such as chemotherapy or molecular-targeted drugs, that activate caspase-3 to cleave GSDME, eventually leading to inflammatory cell death 30, 39, 40. The second way in which GSDME exerts its anticancer effects is through a positive correlation between its expression and the phagocytosis of tumor-related macrophages, as well as the production and function of NK and CD8^+^ T lymphocytes. 31. Granzyme B released by NK cells cleaves GSDME to trigger pyroptosis and simultaneously enhances the function of tumor-infiltrating immune cells, further delaying tumor growth 31. Third, the combination treatment of V-raf murine sarcoma virus oncogene homologue B1 (BRAF) inhibitor and MEK inhibitor promoted the cleavage of GSDME and the release of HMGB1 in melanoma cells, which activated dendritic cells and eventually led to the proliferation of T cells, thus playing an anti-tumor role 41. It is noteworthy that in the mainstream view, PD-L1 inhibitors are believed to primarily impact CD8^+^ T cells to execute their anti-tumor mechanism. Nonetheless, our study proposes that GSDME targets CD4^+^ T cells to achieve its biological function. This significant finding has provided fresh insights, suggesting that future precision immunotherapy may concentrate on modifying CD4^+^ T cells.

SCLC lacks universal predictive biomarkers, such as PD-L1 expression, and the delayed effects of immunotherapy and heterogeneity in SCLC are often underestimated. Pyroptosis-related sensitization to immunotherapy in SCLC provides a novel approach for addressing these challenges. IL-12 is a cytotoxic lymphocyte maturation factor that strongly upregulates cellular immunity. Various methods for local delivery of IL-12, such as plasmid DNA encoding IL-12, mRNA preparations, viral vectors, and fusion proteins, are currently under investigation. These methods have been found to substantially enhance antitumor efficacy and decrease side effects. 42. Our study provides a possible rescue intervention for SCLC patients based on these findings. For SCLC patients lacking GSDME, switching the TME from “cold” to “hot” through chemotherapy may be challenging. However, these patients can be recommended to enroll in relevant clinical trials containing IL-12 to increase their response. Several clinical trials including recombinant IL-12 in solid tumors are currently pending (NCT04025307, NCT00028535). Furthermore, patients with high expression of GSDME tend to show higher sensitivity to chemotherapy, and chemo-rechallenge could be a viable option for clinicians.

Determining the cutoff value of GSDME, which is a potential biomarker for SCLC, is currently a matter of debate as it varies among real-world patients. A recent study showed that epithelial cells with GSDME play a role in the development of colorectal cancer connected with colitis. The study used a score system to determine the percentage of positive cells and staining intensity. However, it did not investigate the connection between GSDME expression and survival 38. A separate study confirmed that the GSDME-YBX1-mucin axis is a unique survival mechanism for patients with pancreatic ductal adenocarcinoma (PDAC). The study determined that the cutoff for GSDME methylation is 50%. Patients with lower levels of GSDME methylation had better survival outcomes 43. In another study, researchers used a semi-quantitative approach to classify patients into two groups based on GSDME expression: GSDME-H (high expression) and GSDME-L (low expression). The study used the staining index (SI) to calculate the proportion of tumor cells multiplied by the staining intensity to determine GSDME IHC's cutoff. If the SI is at least 6, the tissue is classified as having high expression, while an SI below 6 is classified as having low expression 44. Although GSDME has shown potential in clinical applications, there is still a need for additional research to fully understand and define its scope of use in cancer treatments.

The current study has several limitations. Firstly, the changes in the TME resulting from the loss of function due to GSDME mutations and their underlying mechanisms need to be explored further. Secondly, the mechanism upstream of GSDME activation remains unclear, and the role of GSDME methylation in tumorigenesis requires further study.

This study highlights the translational significance of GSDME activation, which triggers rapid inflammatory pyroptosis and induces a subsequent immune response. The use of exogenous IL-12 supplements in GSDME natural deficiency mimics the effect of GSDME activation, thus restoring sensitivity to PD-L1 inhibitors.

## Supplementary Material

Supplementary methods and figures.

## Figures and Tables

**Table 1:** Baseline patient demographics and disease characteristics.

Characteristics	GSDME-low (N=36)	GSDME-high (N=37)	Total (N=73)	P value	FDR
**Age**					
Mean±SD	63.53±7.61	62.00±7.49	62.75±7.53		
Median[min-max]	62.00[53.00,84.00]	62.00[47.00,73.00]	62.00[47.00,84.00]		
**Gender**				0.74	1
Male	33(45.21%)	32(43.84%)	65(89.04%)		
Female	3(4.11%)	5(6.85%)	8(10.96%)		
**Smoking History**				1	1
Yes	29(39.73%)	29(39.73%)	58(79.45%)		
No	7(9.59%)	8(10.96%)	15(20.55%)		
**Stage at diagnosis for SCLC**				1	1
Limited Stage	1(1.37%)	2(2.74%)	3(4.11%)		
Extensive Stage	35(47.95%)	35(47.95%)	70(95.89%)		
PD-L1(%)				0.21	1
1-49	1(1.37%)	5(6.85%)	6(8.22%)		
0	35(47.95%)	32(43.84%)	67(91.78%)		
**Pleura Metastasis**				0.51	1
Yes	8(10.96%)	5(6.85%)	13(17.81%)		
No	28(38.36%)	32(43.84%)	60(82.19%)		
**Brain Metastasis**				0.45	1
Yes	7(9.59%)	11(15.07%)	18(24.66%)		
No	29(39.73%)	26(35.62%)	55(75.34%)		
**Liver Metastasis**				0.7	1
Yes	2(2.74%)	4(5.48%)	6(8.22%)		
No	34(46.58%)	33(45.21%)	67(91.78%)		
**Bone Metastasis**				1	1
Yes	9(12.33%)	10(13.70%)	19(26.03%)		
No	27(36.99%)	27(36.99%)	54(73.97%)		
**Adrenal Gland Metastasis**				1	1
Yes	4(5.56%)	4(5.56%)	8(11.11%)		
No	31(43.06%)	33(45.83%)	64(88.89%)		
