# Supplementary material for: Cisplatin-induced Pyroptosis Enhances the Efficacy of PD-L1 Inhibitor in Small-Cell Lung Cancer via GSDME/IL12/CD4Tem Axis
Source: Int J Biol Sci. 2024 Jan 1;20(2):537–53. doi: 10.7150/ijbs.89080 (PMC10758111; doi:10.7150/ijbs.89080)
Supplement: Supplementary file 1 — Supplementary methods and figures. [file ijbsv20p0537s1.pdf]

Supplementary Materials for

Cisplatin-induced Pyroptosis enhances the efficacy of PD-L1 inhibitor in Small-Cell Lung Cancer via GSDME/IL12/CD4Tem axis

Wendi Xuzhang#, Tingting Lu#, Weiqiu Jin#, Yongfeng Yu, Ziming Li, Lan Shen, Xiaomin Niu, Xinghao Ai, Liliang Xia*, Shun Lu*

#These authors contributed equally to this work.

***Correspondence: Shun Lu (shunlu@sjtu.edu.cn); Liliang Xia (liliangxia@126.com)**

Items	Contents	Pages
Lentiviral infection, Quantitative RT-PCR, Western blotting	Supplementary methods	2
CIBERSORT, Limma Difference Analysis, KEGG enrichment analysis, Immunohistochemistry (IHC)	Supplementary methods	3
Immunofluorescence (IF),Crystal violet staining, Lactate dehydrogenase (LDH) release assay, Western blotting	Supplementary methods	4
Supplementary figure 1	Supplementary figures	5-6
Supplementary figure 2	Supplementary figures	7-8
Supplementary figure 3	Supplementary figures	9-10
Supplementary figure 4	Supplementary figures	11-12
Supplementary figure 5	Supplementary figures	13

Supplementary methods

Lentiviral infection

SHP77, DMS114, and GLC16 cells were seeded onto 12-well plates at 2×10^5 cells/well density. When the cells reached 70% confluence, the medium was carefully removed, and 200 μL of serum-free medium, and virus (multiplicity of infection = 50) were added in this order. After incubation for 24h, the culture medium containing the virus was completely removed, and 1 mL of fresh complete culture medium was added to each well. After 72h of culture, puromycin (2 μg/mL) was added to each well twice to screen stably-infected cells. Infection efficiency was tested using western blotting and qPCR. We generated a GTP-C-3Flag-H-GSDME overexpression plasmid (F:TAGAGCTAGCGAATTCATGTTTGCCAAA GCAACC, R:CTTTGTAGTCGGATCCTGAATGTTCTCTGCCTAA) and CRISPR v2-H-GSDME knockout plasmid (sg-F: TAAGTTACAGCTTCTAAGTC, sg-R: TGACAAAAA AGAAGAGATTC). Corresponding control plasmids were designed. GSDME overexpression (OE) and knockout (KO) lentiviruses were synthesized and packaged by Shanghai Zorin Biological Technology Co., Ltd.

Quantitative RT-PCR

Total RNA was extracted GLC16, SHP77, DMS114 cell lines using RNA-easy Isolation Reagent (Vazyme). Reverse transcription was performed using HiScript III RT SuperMix (Vazyme) according to the manufacturer's instructions. Quantitative real-time PCR was performed using a 7300 AB real-time PCR machine (Applied Biosystems, USA) with 2 × SYBR Green qPCR Master Mix (Servicebio®). The ΔΔCT of genes was normalized to the housekeeping human gene GAPDH. The PCR primers were as follows: GSDME-F: CCCAGGATGGACCATTAAGTGT; GSDME-R: GGTTCCAGGACCATGAGTAGTT; GSDMD-F:

Western blotting

Cells were collected and lysed in RIPA buffer consisting of a phosphatase inhibitor (Roche) and a protease inhibitor (Roche) for 20 min. The protein extracts were electrophoresed in SDS-PAGE and then transferred onto a nitrocellulose filter membrane. The membranes were blocked with 5% skim milk for at least 1 h, washed at least thrice, and incubated in a refrigerator at 4 °C with the primary antibodies, including the anti-caspase-3 antibody (CST), anti-GSDME antibody (Abcam), anti-cleaved caspase-3 (CST), or anti-GAPDH antibody (CST).

CIBERSORT

Based on the database expression profile, we used the Cibersort R software package[34] and calculated the scores of 22 immune infiltrating cells for each sample.

Limma Difference Analysis

We used the R software package Limma (version 3.40.6) to perform differential analysis for obtaining the differential genes between the GSDME high-expression group and the GSDME low-expression group. Specifically, we obtained an expression profile dataset and used the voom function to perform data transformation by removing genes with expression values greater than 50%. Then, we used the ImFit function for conducting multiple linear regression. Empirical Bayesian adjustments were made to the standard error for calculating the logarithmic probabilities of the corrected t-statistic, corrected F-statistic, and differential expression, for obtaining the significant differences of each gene.

KEGG enrichment analysis

For the gene set functional enrichment analysis, we used the KEGG rest API (https://www.kegg. jp/kegg/rest/keggapi.htmL) to obtain the latest gene annotations for the KEGG pathway. The genes were mapped to the background set and enriched using the R software package clusterProfiler (version 3.14.3) to obtain the results of gene set enrichment. The first 20 pathways were selected for drawing. The minimum and maximum genes were set to 5 and 5,000, respectively. All differences were considered to be significant at $p < 0.05$ and FDR < 0.05.

Immunohistochemistry (IHC)

Human samples were obtained from Shanghai Chest Hospital, Shanghai Jiao Tong University School of Medicine (Shanghai, China) after CT-guided percutaneous biopsy or surgical resection. We obtained written informed consent from cancer patients, and they approved the use of tissue samples for research purposes. The Ethics Committee of the Research Institute of Shanghai Jiao Tong University approved this study. We used GSDME (CST) for immunohistochemistry with a 1:200 dilution. Full-slice scanning was performed by Vectra3 (AKOYA BIOSCIENCES), and batch analysis was performed using the inForm software.

Immunofluorescence (IF)

All antibodies were purchased from CST. Multi-fluorescence immunohistochemistry kit-six color TSA-RM-24259 (PANOVUE), including PPD620, PPD520, PPD540, PPD650, and

PPD690 (spectral splitting) were used. The tissue segmenter was trained, and the software automatically learned with an accuracy of over 98%. The following parameters were adjusted to improve the cell resolution, including (a) nuclear signal intensity threshold; (b) sensitivity of the cell nucleus resolution; (c) nuclear size threshold; (d) cytoplasmic thickness. The type of cell was manually defined. A classifier was trained, and the software learned based on the selected feature cells. It classified all cells and continued adjusting until it reached over 99% accuracy. For each image, the software calculated the H-score through positive rate analysis. Individual algorithms were saved and applied to all images, and batch data were integrated.

Crystal violet staining

Human SCLC cell lines were cultivated using a six-well plate. The orifice plate was placed on ice and rinsed twice with pre-cooled PBS. Then, the samples were pre-cooled with 100% methanol (4℃) for 10 min. Methanol was removed from the plate using a pipette. The cells were removed from ice, equilibrated at room temperature, and then incubated for 10 min at room temperature with the crystal violet staining solution for staining. Then, the crystal violet stain was discarded; this crystal violet staining solution could be reused. The cells were washed several times with distilled water until the dye no longer flowed out.

Lactate dehydrogenase (LDH) release assay

We first collected the cell culture supernatant and then measured the activity of LDH using the LDH cytotoxicity assay kit (Abcam) following the manufacturer’s protocol. The LDH levels were normalized in every cell culture supernatant relative to the culture medium in the control group. Each experiment was performed at least thrice.

Supplementary figure 1

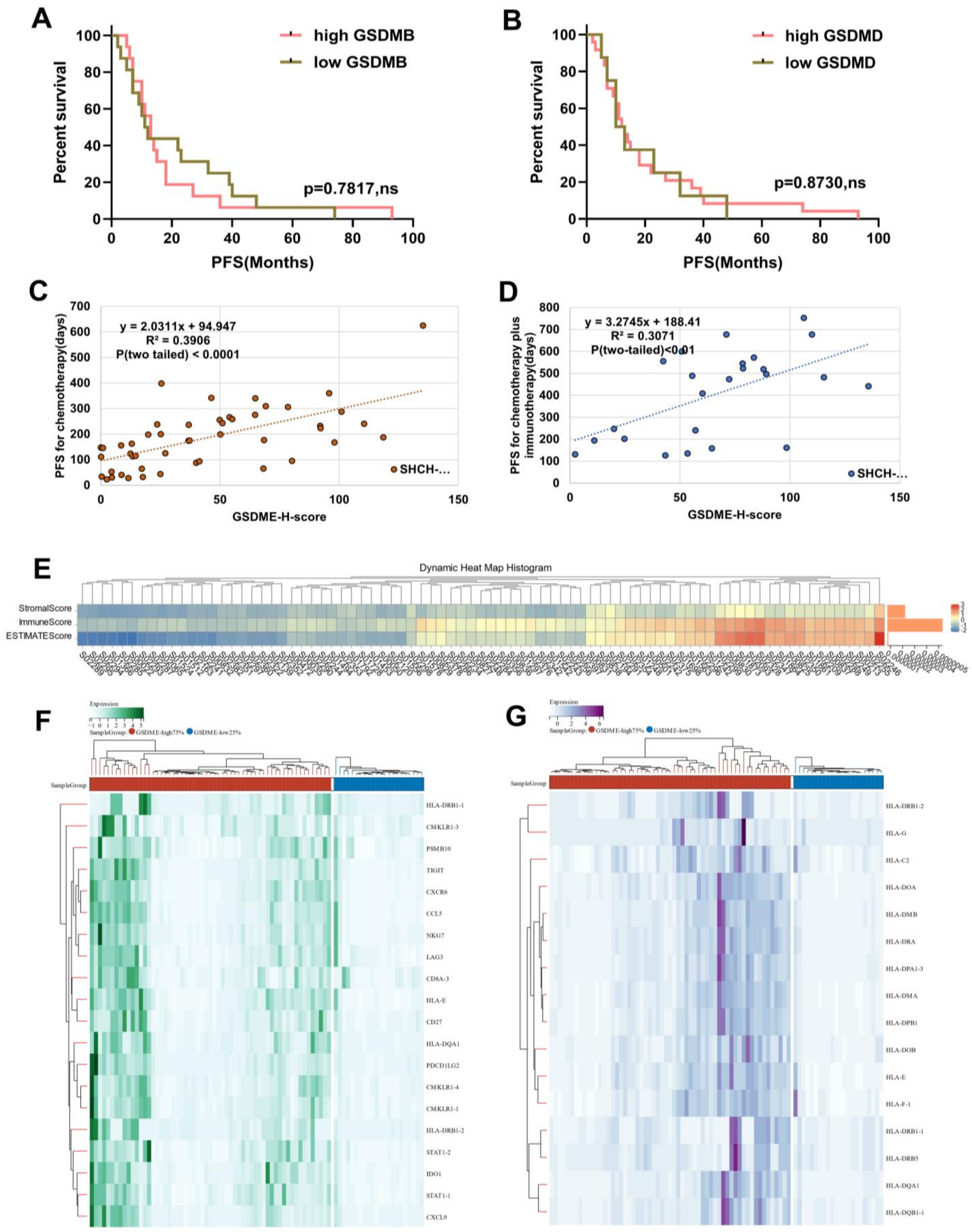

(A) PFS of patients with high GSDMB expression and low GSDMEB expression in small cell lung cancer receiving chemotherapy.
(B) PFS of patients with high GSDMD expression and low GSDMED expression in small cell lung cancer receiving chemotherapy.
(C) The correlation between the H-score of GSDME in tumor tissue and chemotherapy PFS in small cell lung cancer patients.
(D) The correlation between the H-score of GSDME in tumor tissue and the PFS of SCLC patients receiving chemo-immunotherapy.
(E) SCLC patients was arranged from left to right according to the expression of GSDME, and the corresponding SrromalScore, ImmuneScore, ESTIMATEScore was calculated by ESTIMATE, p value was displayed right.
(F) Heat map showed T cell inflammatory related genes in the GSDME-high group and GSDME-low group.
(G) Heat map, showed HLA related genes in the GSDME-high group and GSDME-low group.

Supplementary figure 2

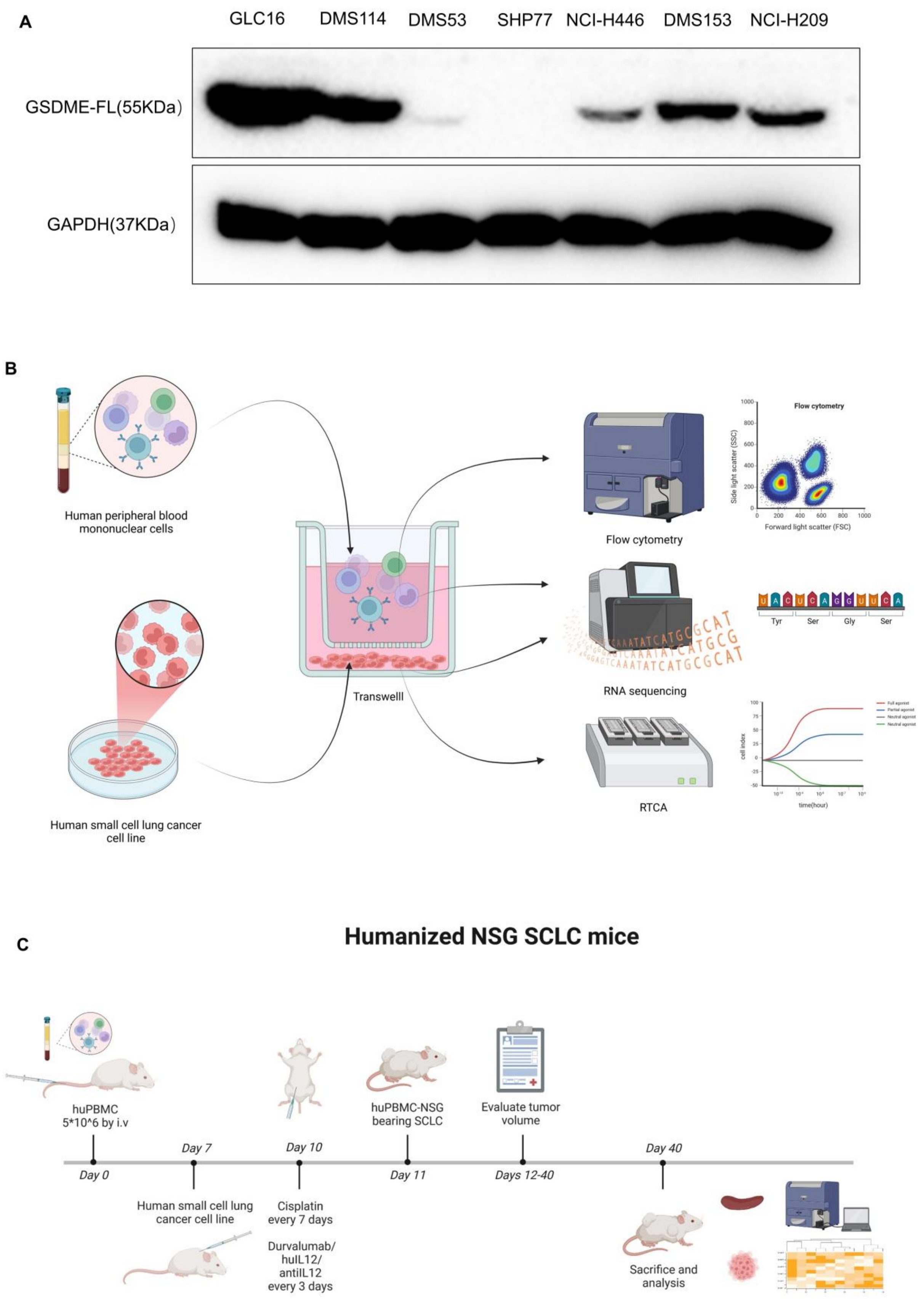

(A) Background expression of GSDME in different human SCLC cell lines.
(B) In vitro co culture model of human SCLC cell and human PBMC. Human small cell lung cancer cells were placed at Transwell's lower chamber, human-derived PBMCs were placed at the upper chamber. Co-culture tumor cells and PBMCs for 48-72h, collect PBMCs and tumor cells using flow cytometry, RNAseq, RTCA, and flow cytometry.
(C) Humanized SCLC NSG mouse model. Firstly, on day 0, 4-6 weeks of NSG female mice were humanized with 5 * 10 ^ 6 PBMCs with huPBMC through the tail vein. On the 7th day, the human SCLC cells were subcutaneously inoculated into the neck and back of humanized NSG mice ($5x10^6$/mouse). On the 10th day or so, the cisplatin/Durvalumab/recombinant IL-12/neutralizing IL-12 antibody was administered according to experimental requirements, and the tumor size was monitored every 3 days for recording. On the 40th day or so, the mice were sacrificed and the tumors were taken for further analysis.

Supplementary figure 3

A

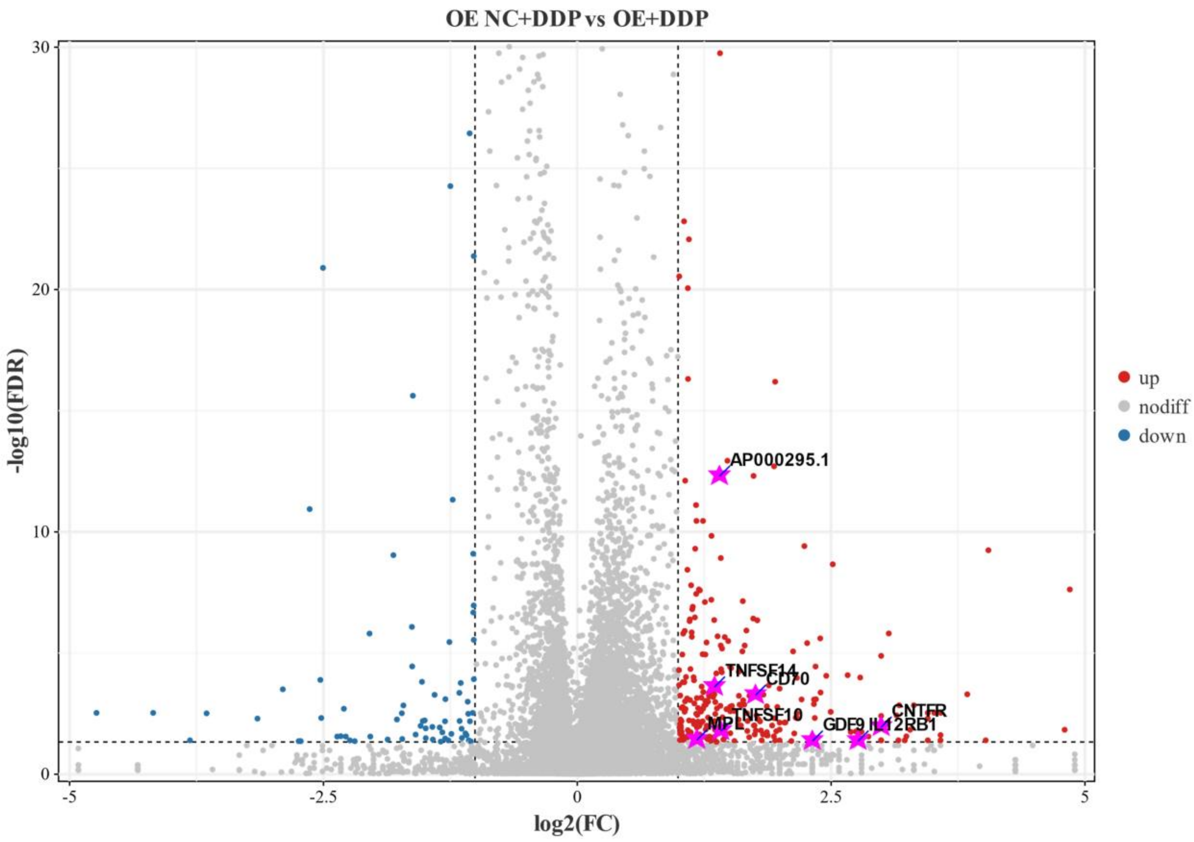

B

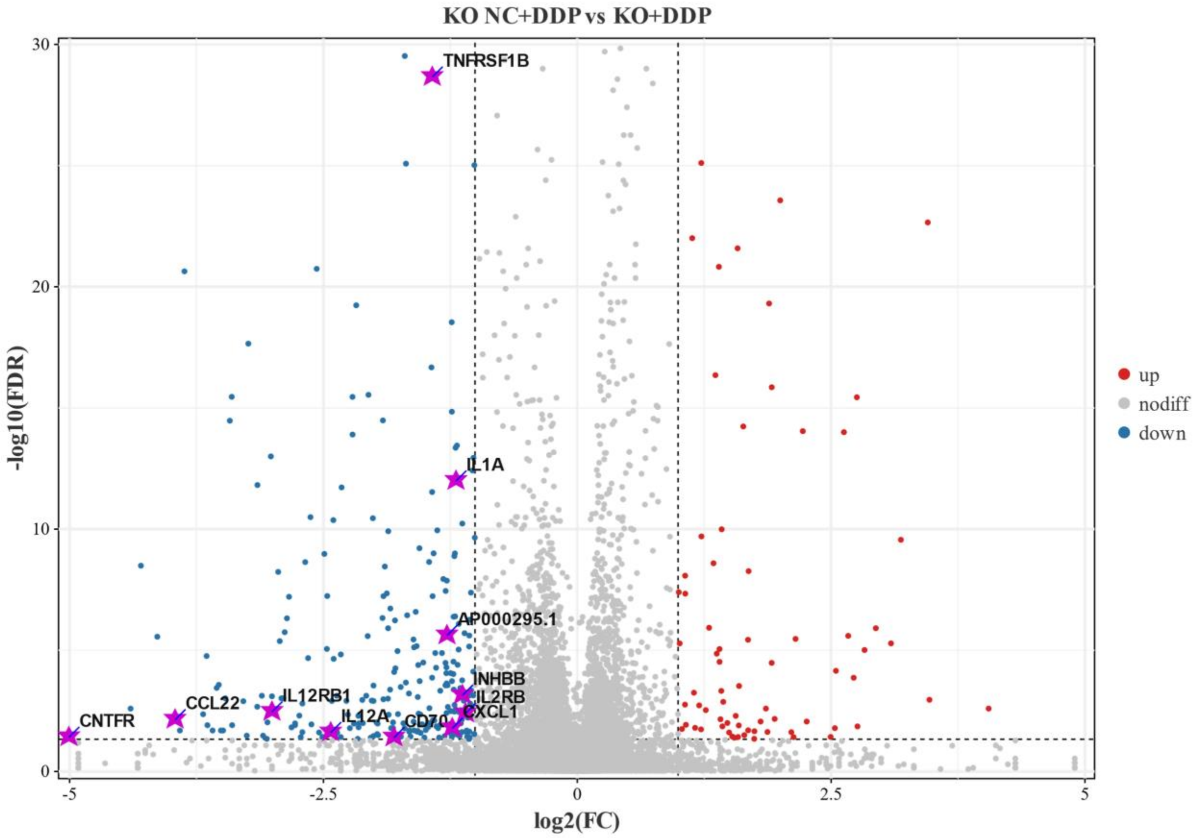

(A) Volcano map showed significantly upregulated genes in the Cytokine-Cytokine receptor interaction (ko04060) pathway in GSDME-OE SCLC cell lines after cisplatin induction.
(B) Volcano map showed significantly downregulated genes in the Cytokine-Cytokine receptor interaction (ko04060) pathway in GSDME-KO SCLC cell lines after cisplatin induction.

Supplementary figure 4

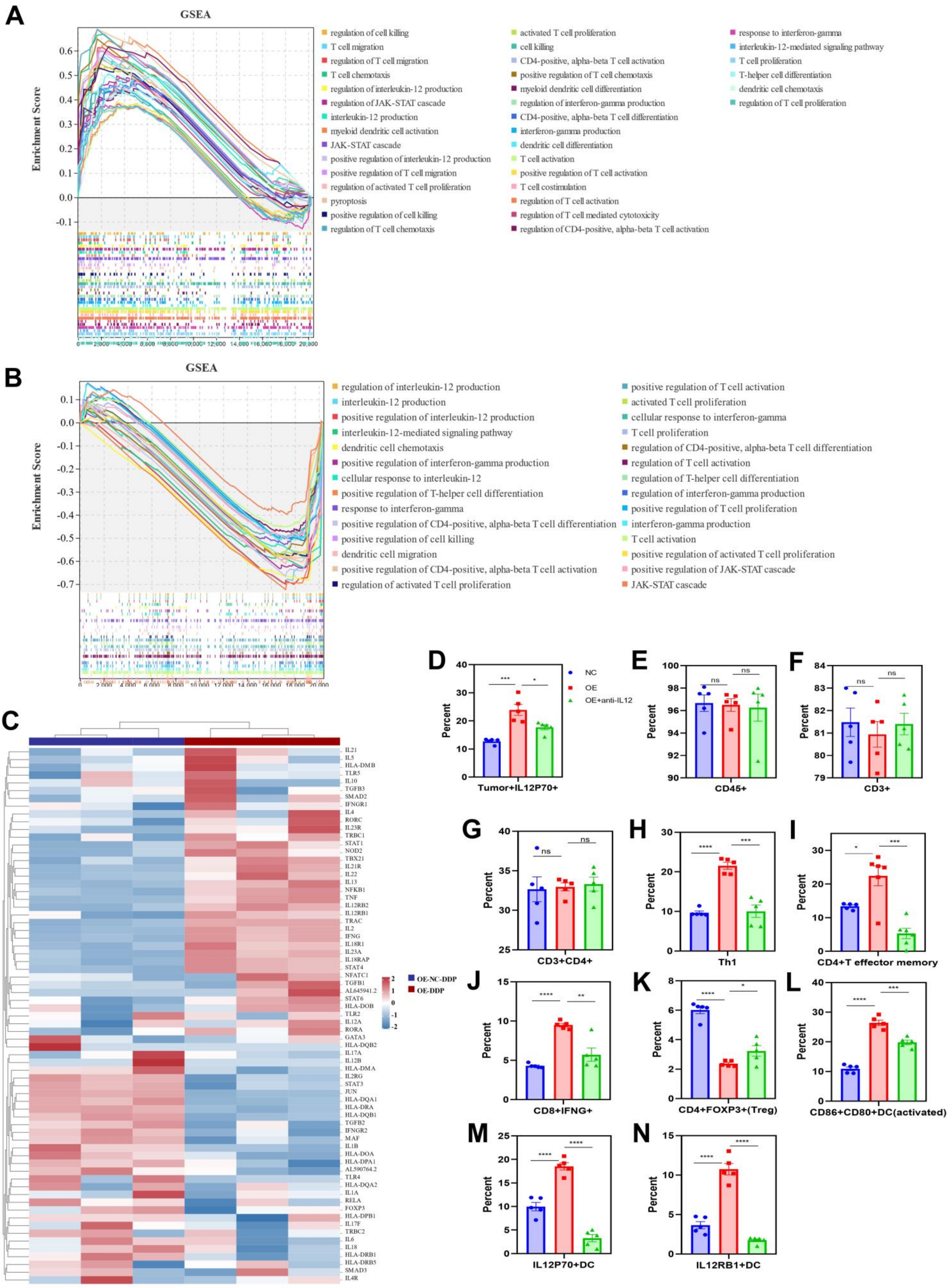

(A) In vitro, GSDME-OE and GSDME-NC DMS114 cells were co-cultured with PBMC, and treated with cisplatin. Cancer cells were collected for RNA-seq. GSEA showed significant upregulated antitumor GO terms involved in GSDME-OE group than in GSDME-NC group.
(B) In vitro, GSDME-KO and GSDME-NC DMS114 cells were co-cultured with PBMC, and treated with cisplatin. Cancer cells were collected for RNA-seq. GSEA showed significant upregulated antitumor GO terms involved in GSDME-OE group than in GSDME-NC group.
(C) In vitro, PBMC was co-cultured with GSDME-OE and GSDME-NC DMS114 cells, treated with cisplatin, and collected for RNAseq, subsequent clustered analysis focus on Th1 and Th2 differentiation pathway was illustrated by heatmap.
(D) In vitro, PBMC was co-cultured with GSDME-OE and GSDME-NC DMS114 cells, treated with cisplatin, and collected for flow cytometry. Tumor IL-12P70(D), CD45+T cell (E), CD3+T cell (F), CD4+T cell (G), Th1 cell (H), CD4+T effector memory cell (I), and CD8+IFN γ+ T cell (J), Tregs (K), activated DCs(L), IL12P70+DC (M), IL12RB1+DC (N) was shown.

Supplementary figure 5

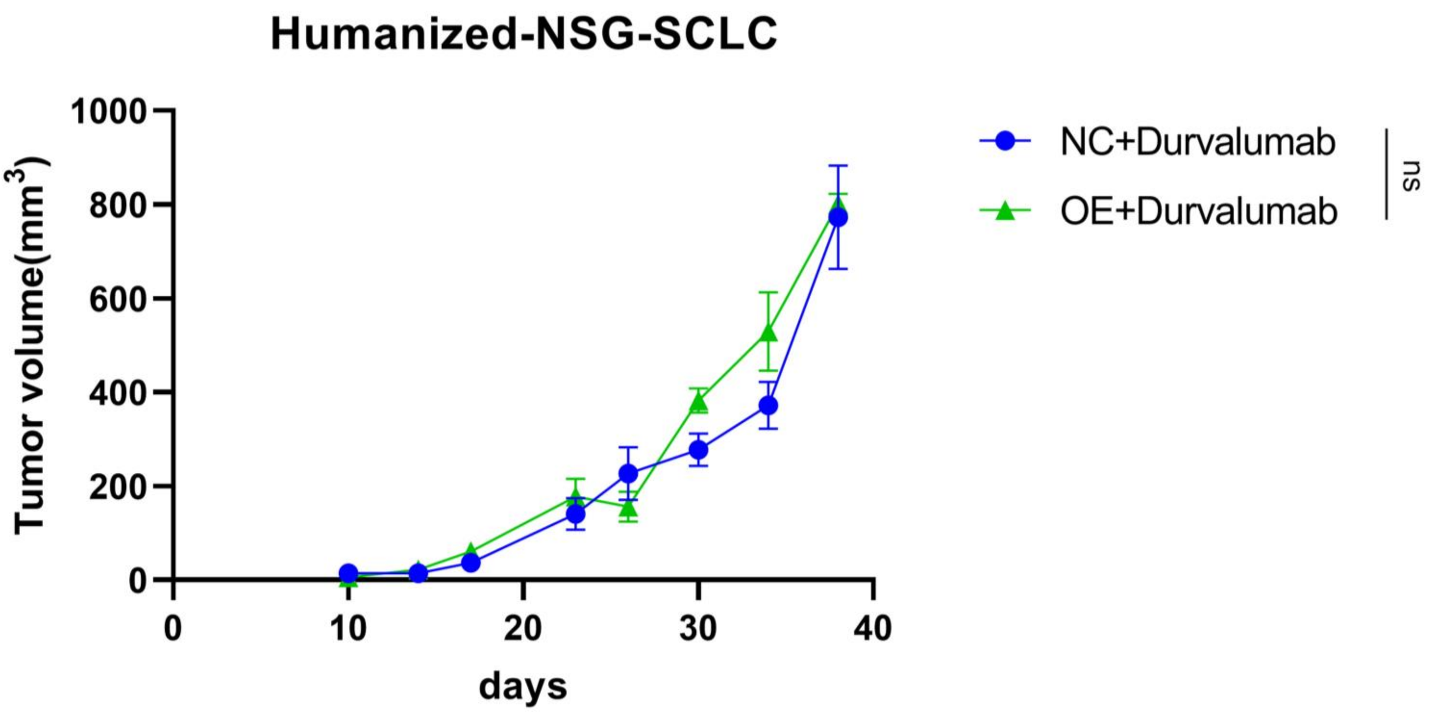

The growth curve of GSDME-OE and GSDME-NC humanized NSG SCLC mice treated with Durvalumab alone(GLC16).
